# Maximal scapular protraction increases the safety distance of the spinal accessory nerve during lower trapezius tendon harvest: a cadaveric study

**DOI:** 10.1016/j.jseint.2026.101622

**Published:** 2026-01-12

**Authors:** Abdul-ilah Hachem, Betlem Fargues, Gonzalo Barraza, Fernando Alvarado, Xavi Rius, Jose Luis Agullo, Diego Gonzalez-Morgado, Bassem Elhassan

**Affiliations:** a Orthopaedic Surgery Department, Hospital Universitari de Bellvitge, L'Hospitalet de Llobregat, Universitat de Barcelona (UB), Barcelona, Spain; b Orthopaedic Surgery Department, University Hospital Arnau de Vilanova, Lleida, Spain; c The Hand & Upper Extremity Center of San Antonio, San Antonio, TX, USA; d Department of Orthopaedic Surgery, Harvard Medical School, Massachusetts General Hospital, MGH Scapula Program, Boston, MA, USA

**Keywords:** Lower trapezius, Tendon transfer, Spinal accessory nerve, External rotation, Scapula, Cadaveric study, Surgical anatomy

## Abstract

**Background:**

The lower trapezius (LT) transfer is a reliable option for treating irreparable posterosuperior rotator cuff tears. The procedure restores external rotation by reproducing the line of pull of the posterosuperior rotator cuff. Despite its proven clinical benefits, the spinal accessory nerve (SAN) remains at risk during LT harvest due to its close relationship with the medial border of the scapula. Moreover, accurate separation of the LT from the middle trapezius (MT) is crucial to obtain the full muscle excursion and optimal mechanical vector of the harvested portion of the muscle. The first aim of this study is to quantify the change in SAN–scapula distance between neutral and maximal scapular protraction positions, and the second aim is to identify a consistent anatomical landmark to differentiate the LT from the MT during tendon harvest.

**Methods:**

Ten fresh-frozen human torsos (20 shoulders) were dissected. Each specimen was positioned in a beach-chair setup at 70°, and the LT and MT insertions were exposed through a posterior approach. The SAN was identified medial to the scapular border and marked. Distances between the SAN and the medial border of the scapula spine were measured in 2 standardized positions by 2 observers and compared: neutral (0° forward flexion, 20° abduction) and maximal protraction (90° forward flexion, 20° adduction). To identify a reproducible landmark distinguishing the LT from the MT, a straight line was drawn from the trapezius tubercle toward the vertebral spinous process, and its vertebral intersection was recorded. For comparison, another line following the axis of the scapular spine was traced to evaluate which orientation excluded proximal LT fibers.

**Results:**

The mean SAN–scapula distance measured 20.4 ± 2.3 mm in the neutral position and increased to 31.8 ± 2.8 mm in maximal protraction, demonstrating a mean increase of 11.4 mm (95% confidence interval, 11.0–11.9; *P* < .001). The SAN was located at a minimum of 17 mm from the medial border of the scapula in neutral position. The reference line from the trapezius tubercle to the vertebral column consistently intersected T3–T4, corresponding to the upper origin of the LT.

**Conclusion:**

Maximal scapular protraction increases the distance between the SAN and the medial border of the scapula, providing a greater margin of safety during LT tendon harvest. In addition, a straight line from the trapezius tubercle to the vertebral spine facilitates clear differentiation of LT fibers from the MT.

The lower trapezius (LT) tendon transfer is a surgical option for the management of irreparable posterosuperior rotator cuff tears, particularly in young and active patients for whom joint-preserving strategies are preferred.^1^^,^^2^^,^^5^^,^^8^^,^^10^^,^^12^^,^^16^^,^^28^^,^^30^ Initially described by Elhassan et al in 2010,^7^ this procedure aims to restore external rotation and improve shoulder function by detaching the LT tendon from its scapular insertion and transfer it to the greater tuberosity of the humeral head, reproducing the line of pull of the infraspinatus and teres minor muscles.^18^^,^^21^^,^^23^ Subsequent clinical series have demonstrated favorable outcomes, including improved range of motion, reduced pain, and high patient satisfaction, with complication rates comparable to other tendon transfers.^1^^,^^4^^,^^8^^,^^10^^,^^17^^,^^28^^,^^32^

The spinal accessory nerve (SAN) represents the most critical structure at risk during LT harvest. The SAN courses deep to the trapezius and lies in close proximity to the medial border of the scapula, particularly near the upper portion of the LT.^13^^,^^20^ Accurate anatomical knowledge is therefore essential to avoid iatrogenic injury, as nerve damage may result in trapezius weakness and scapulothoracic abnormal motion.^3^^,^^11^^,^^25^ Despite these risks, few anatomical studies have provided quantitative guidelines to define a safe dissection zone for LT harvest.^13^^,^^20^ Elhassan et al^9^ reported that the SAN is 2 cm medial to the medial border of the scapula. Whereas Gracitelli et al^13^ measured the nerve 3 cm from the medial border of the scapula in maximum protraction. These observations suggest that scapular protraction could increase the safety margin between the SAN and the scapula, although this hypothesis has not been validated.

Another key challenge during LT harvest is the accurate distinction between the middle trapezius (MT) and LT. Harvesting only part of the LT or inadvertently including MT fibers may compromise the tendon's excursion and mechanical vector, potentially leading to suboptimal function of the transfer.^31^ Omid et al^20^ described the LT as a flat triangular tendon inserting at the dorsal trapezius tubercle, while the MT inserts more broadly along the superior surface of the scapular spine. However, there remains limited guidance on how to include all LT fibers along their medial extent, creating uncertainty regarding the optimal dissection trajectory to encompass the entire muscle belly. Precise differentiation and complete inclusion of LT fibers are essential to maximize the muscle's physiological potential and ensure an effective transfer.

Therefore, the primary objective of this study was to quantify the change in SAN–scapula distance between neutral and maximal scapular protraction positions. The secondary objective was to identify a reproducible anatomical landmark for differentiating the MT and LT muscles during tendon harvest. We hypothesized that maximal scapular protraction would significantly increase the distance between the SAN and the medial border of the scapula, and that a straight line drawn from the dorsal trapezius tubercle toward the vertebral column would encompass the entire length of the LT.

## Methods

### Specimens and study design

This study adhered to QUACS (Quality Appraisal for Cadaveric Studies) guidelines. Ten fresh-frozen human torsos, including both upper limbs and with no history of shoulder surgery, were obtained from the Body Donation Service and Dissecting Room at the Bellvitge Hospital Campus (University of Barcelona). All dissections were performed by a single surgeon (B.F.P.) under the supervision of the senior author (A.H.). Measurements were obtained independently by 2 surgeons (A.H. and F.A.) using a digital hand-held caliper.

### Cadaveric dissection

Specimens were positioned in a beach-chair setup at 70° of inclination. A longitudinal skin incision was made from the inferior border of the acromion to the midpoint between the medial border of the scapula and the vertebral column, extending to the inferior scapular angle and returning to the subacromial region. This exposure provided direct visualization of the inferior MT, LT, and their insertions on the scapular spine.

To distinguish MT from LT fibers, 3 criteria were applied: (1) orientation of the muscle fibers; (2) insertion characteristics and location; and (3) changes in fiber tension during maximal scapular protraction. The MT fibers have a transverse orientation,^14^ and insert horizontally and broadly along the dorsal surface of the scapular spine, consisting predominantly of muscle belly with minimal tendon.^20^ The most inferior MT fibers insert at the dorsal trapezius tubercle on the dorsal surface of the scapular spine. The LT forms a flat triangular tendon arising from the medial border of the scapula and converging toward the dorsal trapezius tubercle on the dorsal surface of the scapular spine.^20^ Maximal scapular protraction consistently increases LT tension, enabling clear identification of its fibers.^13^

**Figure 1:**
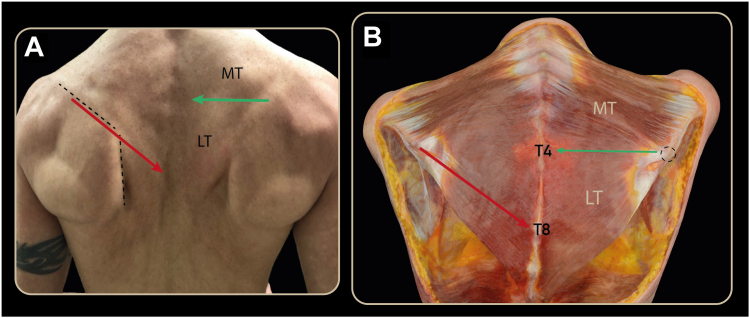
(**A**) Superficial landmarks for tendon harvesting: scapular spine and medial border of the scapula (*dashed line*). (**B**) A line drawn from the trapezius tubercle (*dashed circle*) to the corresponding vertebral spinous process intersects T3-T4 (*greenarrow*), corresponding to the Upper origin of the Lower trapezius. A line drawn in continuation with the axis of the scapular spine (*red arrow*), intersects T7–T8 and excludes proximal Lower trapezius fibers. *MT*, Middle trapezius; *LT*, Lower trapezius.

To establish a reproducible anatomical landmark distinguishing the LT from the MT, a straight line was drawn from the trapezius tubercle toward the corresponding vertebral spinous process with the scapula in maximal protraction. This line consistently intersected T3–T4, corresponding to the upper origin of the LT. For comparison, another line was drawn in continuation with the axis of the scapular spine, and its vertebral intersection was determined. This line intersected T7–T8 and excluded proximal LT fibers according to the predefined criteria for differentiation between MT and LT (Fig. 1).

**Figure 2:**
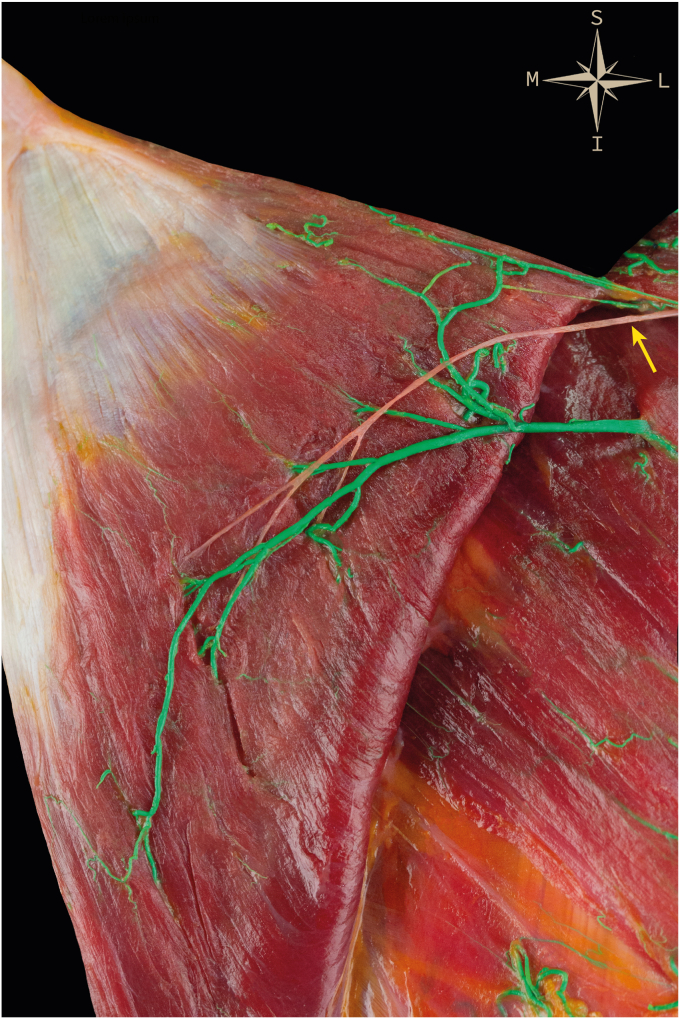
*Lower* trapezius muscle detached from the scapula and reflected medially, exposing its ventral surface and showing the spinal accessory nerve (*yellow arrow*) and the superficial branch of the transverse cervical artery (*green arrow*).

**Figure 3:**
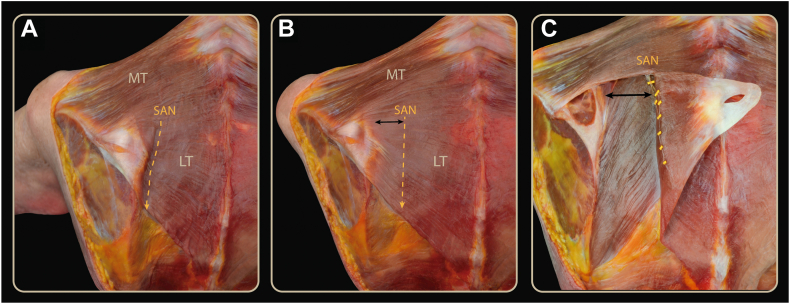
(**A**) With the scapula in a neutral position, the spinal accessory nerve is located 1–2 cm from the medial border of the scapula. (**B**) Scapula in maximum protraction, showing an increased distance between the spinal accessory nerve (*yellow dashed arrow*) and the medial border of the scapula (*black arrow*). (**C**) Scapula in maximum protraction with the lower trapezius detached, showing the distance (*black arrow*) between the medial border of the scapula and the spinal accessory nerve (*yellow marker*). *MT*, Middle trapezius; *LT*, Lower trapezius; *SAN*, spinal accessory nerve.

Following identification of the dorsal trapezius tubercle and LT tendon, a straight deep incision was performed along the line from the tubercle to the medial border of the scapula. Once the medial border of the scapula was reached, a superficial incision along this trajectory exposed the muscle belly and underlying deep fascia. Approximately 1–2 cm from the medial border, blunt dissection was used to locate the SAN (Figs. 2 and 3). It is worth noting that the distance between the SAN and the medial border of the scapula varies along its course, being shorter proximally and increasing progressively distally. The LT was then detached from the medial border of the scapula, dissected across the rhomboid major fascia, and followed until reaching the fat pad containing the nerve on its anterior surface (Fig. 3).

### Measurements

The cadaveric arm was mounted on a support system (Trimano Fortis Support Arm; Arthrex, Inc., Naples, FL, USA). This allowed a reliable surgical setup and control of scapular motion. The SAN was dissected and marked with a yellow marker.

**Figure 4:**
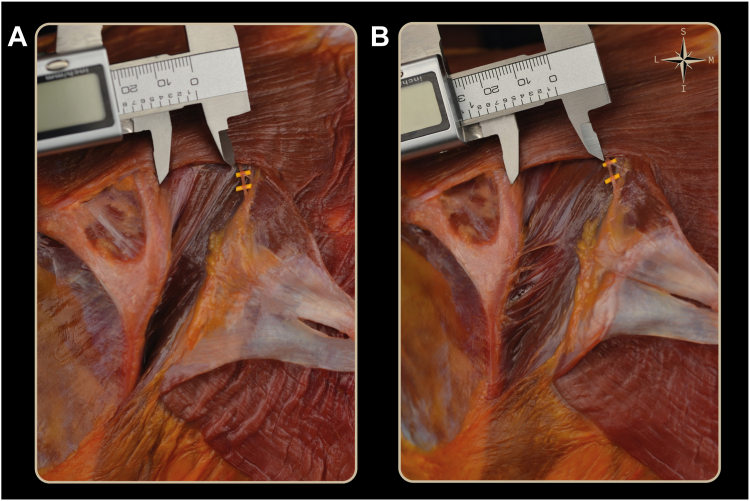
Measurements between the spinal accessory nerve (*yellow marker*) and a point located 1 cm proximal to the superomedial border of the scapular spine. (**A**) Neutral position, showing an approximate distance of 2 cm. (**B**) Maximum protraction, showing an increase of approximately 1 cm.

**Figure 5:**
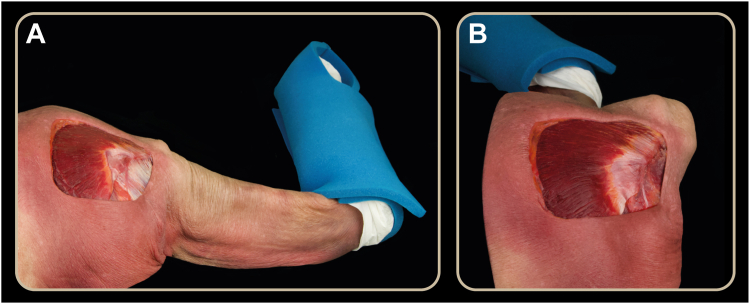
(**A**) Neutral position (0° forward flexion and 20° abduction) (**B**) Maximum protraction (0° forward flexion and 20° adduction).

Distances were measured by the 2 surgeons for each shoulder from 1 cm proximal to the superior–medial border of the scapular spine to the identified SAN along a straight line directed toward the corresponding spinous process. The observers were blinded to each other's assessments. Measurements were performed in 2 standardized positions: (1) *neutral*: 0° forward flexion, 20° abduction and (2) *maximal protraction*: 90° forward flexion, 20° adduction (Fig. 4). The transition between positions was obtained by forward flexing the arm with the Trimano device while rotating the torso until maximum adduction was achieved (Fig. 5).

### Statistical analysis

Data distribution was assessed with the Shapiro–Wilk test to verify normality. Continuous data were reported as a mean with standard deviation and range. The average distance measured by the 2 surgeons for each shoulder, in both the neutral scapular position and maximal protraction, was calculated to determine the mean distances for each cadaver. To assess interobserver reliability, Cohen's kappa coefficient was calculated for each condition. A 2-sided paired Student's t test was applied to compare the SAN – dorsal trapezius tubercle distance in the neutral and in the maximal protraction positions. Differences were reported as mean difference with corresponding 95% confidence intervals (CIs). A *P* value <.05 was considered statistically significant. All statistical analyses were conducted using SPSS version 25 (IBM Corp., Armonk, NY, USA).

## Results

The mean age of the specimens was 82.8 years. Four specimens (80%) were female. Interobserver agreement for the measurement was substantial under both conditions, with kappa values of 0.796 for the neutral scapular position and 0.783 for maximal protraction. The mean SAN – dorsal trapezius tubercle distance in the neutral position was 20.4 ± 2.3 mm (range 17.2 – 23.8 mm). In maximum protraction, the distance increased to 31.8 ± 2.8 mm (range: 28.1 – 36.7 mm), with a mean difference of 11.4 mm (95% CI [11.0 – 11.9; *P* < .001]). Table I shows the measurements of the SAN–scapula distance.

**Table I:** Measurements of the spinal accessory nerve–scapula distance.

Specimen - shoulder	Distance in neutral (mm)	Distance in maximal protraction (mm)
1 – Right	22.5	34.5
1 – Left	17.4	29.7
2 – Right	22.3	33.6
2 – Left	20.3	33.1
3 – Right	17.8	28.2
3 – Left	20.2	30.6
4 – Right	23.8	36.2
4 – Left	22.8	34.6
5 – Right	17.9	28.5
5 – Left	18.9	29.6
Mean ± Standard deviation	20.4 ± 2.3	31.9 ± 2.8
Mean difference	11.4 (95% CI [11.0 – 11.9; *P* < .001])	

*CI*, confidence interval.

Regarding reproducible anatomical landmarks to distinguish the LT from the MT, a straight line traced from the trapezius tubercle to the corresponding vertebral spinous process consistently intersected T3–T4, corresponding to the upper origin of the LT. In contrast, a line drawn in continuation with the scapular spine intersected T7–T8, excluding proximal LT fibers, according to the established criteria for distinguishing the fibers of MT and LT.

## Discussion

This cadaveric study demonstrated that the distance between the superomedial border of the scapula and the SAN increases by approximately 1 cm when the scapula is moved from a neutral to a maximally protracted position. These findings suggest that scapular protraction may enhance intraoperative safety during LT transfer. Additionally, a reproducible anatomical landmark reference to distinguish the LT from the MT was identified: a straight line drawn from the dorsal trapezius tubercle to the vertebral column consistently intersected T3–T4, marking the superior limit of the LT and enabling reliable distinction from the MT.

Previous anatomical studies have emphasized the vulnerability of the SAN during LT harvest due to its close relationship to the medial border of the scapula.^13^^,^^20^ Omid et al^20^ reported that the SAN is closest to the LT tendon at the superior border of the scapula and gradually moves away distally, with distances ranging from as little as 23 mm to an average of 58 mm.

In our series, the closest nerve was identified at 17 mm from the medial border of the scapular spine, highlighting an even narrower margin of safety. It should be noted, however, that Omid et al^20^ measured from the tendon insertion, which limits direct comparison with our method. Gracitelli et al^13^ measured the SAN–scapula distance in maximal protraction at an average of 32.5 mm, which aligns with our finding that protraction increases the distance by approximately 1 cm. This additional margin may be clinically relevant, as it reduces the likelihood of inadvertent SAN injury and supports the recommendation to perform LT dissection with the scapula in maximal protraction.

Although the SAN remains the most critical structure at risk during LT tendon harvest, clinical evidence consistently indicates that true iatrogenic SAN injury is extremely rare. To date, no published study has reported a case of SAN palsy following LT transfer. The most frequently reported complications are seroma formation, infection, hematoma, and graft failure rather than neural injury.^4^^,^^17^^,^^29^ Elhassan et al^8^ described 4 cases of transient hand numbness along the thumb or ulnar nerve distribution related to postoperative orthotic compression rather than intraoperative injury, resolving spontaneously within 1 to 3 months after brace removal. Other clinical series have also reported no cases of SAN injury despite technical variations in graft type and fixation method.^1^^,^^26^^,^^28^^,^^32^ Notably, Woodmass et al^32^ documented an axillary nerve palsy in a latissimus dorsi transfer cohort for massive posterosuperior rotator cuff tears but none in the LT group, emphasizing the neurological safety profile of LT transfer.

Nevertheless, the risk of nerve injury during dissection should not be ignored. Gracitelli et al^13^ reported that in 11 of 12 cadaveric dissections extending the LT medially toward its origin, SAN injury occurred, underscoring that medial dissection beyond the scapular border places the nerve at risk. This finding reinforces the relevance of scapular protraction, which increases the distance between the SAN and the medial border of the scapula and minimizes the potential for inadvertent nerve damage during LT harvest.

The absence of reported iatrogenic SAN injuries during LT harvest can be attributed to several factors. First, most LT transfers may be performed by surgeons experienced in complex shoulder surgery, who are well aware of the regional anatomy and the need to respect safe dissection planes. Second, transient neurapraxias may go unnoticed during postoperative immobilization or may not result in evident trapezius dysfunction due to the muscle's dual innervation by the cervical plexus.^15^^,^^19^^,^^22^^,^^27^ The SAN provides the primary motor supply, but additional motor fibers arise from the cervical plexus (C2–C4), which may mitigate the clinical expression of incomplete SAN injuries.^15^^,^^19^^,^^22^^,^^27^

Accurate identification of the LT and MT is essential during tendon harvesting to optimize the function of the fully transferred lower trapezius and avoid harvesting the MT which is essential for the scapula stability and functionality. Classical anatomical texts provide limited guidance on trapezius subdivisions at the scapular level, offering only general descriptions of the muscle's attachments and orientation.^24^ Gracitelli et al^13^ analyzed the feasibility of transferring different portions of the trapezius but did not specify criteria for distinguishing the LT from the MT. Elhassan proposed that the optimal way to identify the interval between the LT and MT is to dissect medially along the superior border of the triangular tendinous portion of the LT insertion toward the vertebral spine.^6^^,^^10^ Omid et al^20^ described the LT as originating from T1–T3 through T11–T12, with its insertion located at the dorsal trapezius tubercle. In their cadaveric dissections, the authors introduced the so-called hook technique, in which the surgeon “hooks” the index finger laterally beneath the trapezius and over the smooth triangular area to grasp the dorsal lower fibers of the LT.^20^ This technique is useful for distinguishing the LT tendon and its adjacent muscular fibers near the insertion, helping to extend the dissection medially or for establishing clear vertebral-level orientation.

The present study expands on previous anatomical descriptions,^6^^,^^10^^,^^20^ by demonstrating that a straight line drawn from the trapezius tubercle toward the vertebral column consistently intersects T3–T4, defining the superior boundary of the LT. In contrast, extending the dissection along the scapular spine excludes proximal LT fibers. This reproducible landmark facilitates differentiation from the MT and guides the medial extent of dissection. From a practical standpoint, this T3-T4 level can be marked on the skin preoperatively to provide a simple external reference that helps orient the medial extent of the dissection intraoperatively. Moreover, maximal scapular protraction increases LT tension while leaving the MT relatively slack, providing an additional visual and tactile cue for intraoperative identification. These anatomical refinements enhance surgical precision and minimize the risk of harvesting MT fibers or omitting proximal LT fibers.

This study provides quantitative evidence that maximal scapular protraction significantly increases the distance between the SAN and the medial border of the scapula, supporting a simple and reproducible intraoperative maneuver to enhance safety during LT harvest. However, several limitations should be acknowledged. The advanced age of the cadaveric specimens may not fully represent the anatomy or tissue properties of younger clinical populations. In addition, the presence of kyphosis in 2 specimens likely limited scapular mobility, potentially underestimating the observed increase in distance. The cadaveric nature of the study also precludes evaluation of intraoperative factors such as muscle tone, soft-tissue elasticity, and patient positioning under anesthesia. Finally, the use of isolated torsos rather than whole bodies restricted specimen fixation, preventing complete replication of operative conditions.

## Conclusion

Maximal scapular protraction increases the distance between the SAN and the medial border of the scapula, providing a greater margin of safety during LT tendon harvest. In addition, a straight line from the trapezius tubercle to the vertebral spine facilitates clear differentiation of LT fibers from the MT.

## Disclaimers:

Funding: No funding was disclosed by the authors.

Conflicts of interest: Abdul-ilah Hachem declares consulting fees from Arthrex and Stryker and royalties from Arthrex. Bassem Elhassan declares consulting fees and royalties from DJO. The other authors, their immediate families, and any research foundation with which they are affiliated have not received any financial payments or other benefits from any commercial entity related to the subject of this article.
